# Special Issue: Antibody Therapy for Hematologic Malignancies

**DOI:** 10.3390/ijms26199463

**Published:** 2025-09-27

**Authors:** Jonathan P. Butchar

**Affiliations:** Division of Hematology, The Ohio State University Wexner Medical Center, 410 W, 12th Avenue, Columbus, OH 43210, USA; jon.butchar@osumc.edu; Tel.: +1-614-366-0726

It was recently reported that hematologic malignancies represented 6.6% of total cancer cases and that the global lifetime risk of incidence was 1.67% in 2022 [1]. The economic burden was estimated at roughly 1% of global GDP in 2021 [2]. Quite expectedly, there have been great efforts toward creating therapies against these cancers. This has led to a wide variety of treatments, with new agents and modalities emerging on an essentially perpetual basis. Inhibitors of Bruton Tyrosine Kinase (BTK) are used for B-cell malignancies and continuously evolve to address resistance [3,4]. Chimeric antigen receptor (CAR) T cell therapy has shown effectiveness [5] and universal CAR T (UCAR-T) (derived from allogeneic healthy donors) has been making great strides [6]. Checkpoint inhibitors are in use and testing [7], vaccines continue to be developed [8] and PEGylated/engineered cytokines are being studied [9]. We also see continued advances with combination therapies [10,11,12,13,14].

Among these developments, there has been substantial growth of antibody-based treatments. Rituximab, approved by the US Food and Drug Administration (FDA) in 1997 [15], represented the first such agent and has shown efficacy in a range of B-cell malignancies [16]. It is a chimeric antibody consisting of murine anti-CD20 variable regions with human IgG1 and κ light chain constant regions [17]. Rituximab leads to B-cell depletion by binding CD20 on the B cells and triggering complement-dependent cytotoxicity (CDC) as well as antibody-dependent cellular cytotoxicity (ADCC) [17] and apoptosis [18,19,20]. Rituximab has now been joined by a large number of additional antibodies, from engineered anti-CD20 antibodies such as obinutuzumab [21] to cytokine-antibody fusions [22] and bispecific antibodies [23]. This Special Issue focuses on recent advances made with antibody therapy, highlighting key developments and summarizing a battery of clinical trials for leukemias and lymphomas.

Lymphoid malignancies have generally been responsive to antibody therapy, and this is reviewed by Naman et al. [16]. They provide an overview of the major types of antibodies (Figure 1). Starting with rituximab, they discuss the design, production and clinical applications of antibodies for hematologic malignancies, as well as adverse effects and resistance mechanisms. Since the first antibody, severe effects have been seen such as acute tumor lysis syndrome [24,25,26]. Indeed, even with the later-generation antibody obinutuzumab, adverse events including infusion-related reactions, thrombocytopenia and cardiac-related effects have been seen [27], along with rare but more serious severe acute thrombocytopenia [28]. As pointed out by Naman et al., however, most adverse events have been well managed over the years.

Naman et al. also address the critical topic of resistance to antibody therapy, which was documented shortly after the approval of rituximab when loss of the CD20 antigen was seen in some relapsing patients [29,30]. This antigen loss is discussed, along with other resistance mechanisms. Following this, they move forward with engineered antibodies, ADCs and bispecific antibodies. They then highlight treatment strategies, paying particular attention to combination therapy. This can entail two different antibody-based agents, an antibody plus a non-antibody drug such as lenalidomide or an antibody with a checkpoint-targeting agent. The potential for artificial intelligence (AI) to substantially impact the development of antibodies via prediction of high-immunogenicity targets is also discussed.

One factor associated with treatment resistance across multiple cancers is c-MYC overexpression [31]. To explore this in greater detail, de Jonge et al. [32] in this Special Issue used inhibitor and CRISPR approaches to identify mechanisms by which MYC could promote resistance. They found a combination of responses affected by MYC such as antigen presentation, expression of apoptosis-related proteins and effects on T-cell activation. This paves the way for multiple therapeutic approaches, which are discussed by the authors.

Roughly forty years ago, bispecific antibodies were demonstrated as a potential means of directing T cells against specific tumor-associated targets [33,34]. Here, the antibodies were created by protein crosslinking or by hybrid hybridomas. Advances in technology have greatly facilitated the generation of these antibodies, with numerous production platforms available [35]. Perhaps the most well-known bispecific antibody today is the fragment-based bispecific T-cell engager (BiTE) blinatumomab, showing great success against acute lymphocytic leukemia (ALL) [36,37]. For CLL, a relatively recent development has been a bispecific antibody targeting the aberrant IGLV3-21^R110^ B-cell receptor (BCR) and CD3 [38]. The same group had previously developed CAR T cells targeting the IGLV3-21^R110^ BCR, with the bispecific antibody created as a possible therapy for patients unable to tolerate CAR-T treatment [39].

In this Special Issue, Amoozgar et al. [40] provide a rich review of bi- and tri-specific antibodies that includes design, mechanisms and targets (Figure 2) and their efficacies in various leukemias and lymphomas. They then discuss adverse events and resistance mechanisms commonly seen with the use of these antibodies. Several interesting potential future directions are also mentioned, including multispecific constructs that incorporate immune modulators or checkpoint inhibitors. The latter is likely to be highly useful for CLL patients, as PD-1 expression on CLL cells is dependent on BTK and is higher in patients showing disease progression while receiving BTK inhibitor therapy [41]. Amoozgar et al. also discuss masked constructs that are cleavable by tumor-specific protease-sensitive linkers, potentially maximizing on-target effects and reducing toxicities. Finally, they delve into the world of AI, discussing AI-guided antibody design, the prediction of patient responses antibodies and treatment optimization.

For bispecifics in general, much of the focus has been on αβ T cells but γδ T cells have also been intensively investigated. These cells are MHC-independent, expandable and highly cytotoxic (reviewed in [42]). In a research article within this Special Issue, Marischen et al. [43] examined the effects of the anti-CD3/CD20 bispecific antibody mosunetuzumab on γδ T cells, comparing against obinutuzumab. Using a series of co-culture experiments, they found that mosunetuzumab was more effective at activating γδ T cells and driving stronger γδ T cell-mediated elimination of cell-line and primary-cell targets. Due to the expandable nature of γδ T cells and the established safety profile of mosunetuzumab [44,45], combined mosunetuzumab plus stimulated γδ T cells may represent a practical and powerful therapeutic modality. The authors discuss this, as well as the possibility of testing γδ T cells against additional bi- and tri-specific antibodies, which would make this approach more generalizable.

As mentioned by both Naman et al. and Amoozgar et al., safety remains a concern with bispecific antibodies. To gain a clearer understanding of this, Bayly-McCredie et al. [46] used a structured systematic review approach to evaluate the safety and efficacy of bispecific antibodies for large B-cell lymphomas (LBCLs), comparing against CAR-T therapy. Results showed a tradeoff, with bispecifics having good efficacy and tolerability and CAR-T therapy having greater efficacy with less tolerability. This not only provides impetus for further refinement of bispecifics, it indirectly hints at the contribution of economic and logistical factors in the choice of therapeutic modality, an idea brought up by Juarez-Salcedo et al. [47] in this Special Issue.

In their review, Juarez-Salcedo et al. focus on the antibody-drug conjugate, loncastuximab tesirine, as a treatment for diffuse large B-cell lymphoma (DLBCL). Here, humanized anti-CD19 is linked to SG3199, a DNA-targeting alkylating agent. Before and after its approval by the US Food and Drug Administration in 2021 [48], this ADC has received continuous attention in clinical trials. The authors discuss these trials, past and ongoing. Current trials include pairing loncastuximab with other antibodies such as rituximab, glofitamab, mosunetuzumab and polatuzumab vedotin. These combinations offer the advantage of targeting two different antigens on the tumor cells and of combining two methods of killing. Loncastuximab leads to tumor-cell death by binding DNA, while biscpecific anti-CD20 glofitamab [49] and mosunetuzumab [50] recruit T cells and the anti-CD79B ADC polatzumab vedotin disrupts microtubule formation [51]. We anxiously await the results of these trials.

Despite advances such as those reviewed above, there are still hematologic malignancies not yet fully amenable to these therapies. Damiani and Tiribelli [52] discuss a major such disease, acute myeloid leukemia (AML). They highlight several contributing factors such as difficulty identifying good target antigens, low mutational burden and low immunogenicity of candidate antigens. Despite this, antibodies are in use and testing that target antigens such as CD33, CD123 and CD371. The authors discuss these and additional antibodies, presented with a useful background section on antibody therapy in general. Specific antibodies are evaluated in ample detail, highlighting their design, targets, efficacy and caveats. Finally, the authors discuss several potential ways to improve antibody therapy for AML. These include surfaceome analysis, the use of multiple binding sites, engineered antibodies that are activated only when all targets are engaged, overcoming the immunosuppressive environment, and other interesting ideas.

Antibody therapy plays a key role in the treatment of hematologic malignancies, and antibody-based modalities will certainly improve over time as biological and computational technologies advance. Malignant cells are well known for their ability to escape both non-antibody-based [53] and antibody-based [30] treatments, so this continued progress is greatly needed. On behalf of the authors, we hope you find this Special Issue enjoyable and useful.

## Figures and Tables

**Figure 1:**
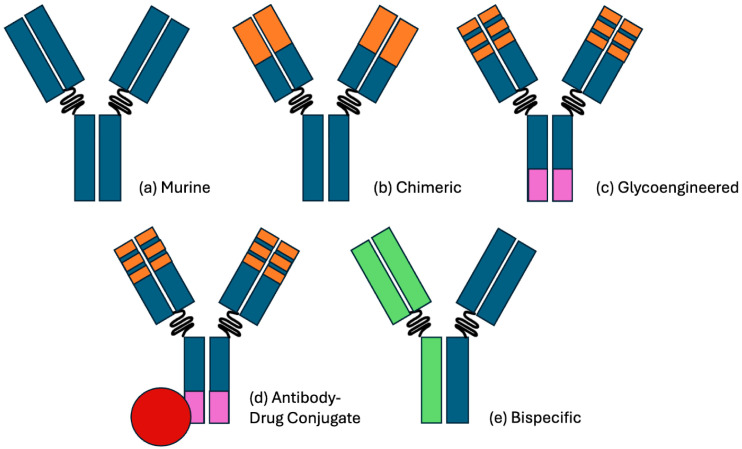
Visual representation of the antibody therapy classes. (**a**) A pure murine antibody, as derived from hybridoma cell lines. (**b**) A chimeric antibody with humanized (orange) fAb regions, improving the immunogenicity and half-life. A notable example is rituximab, which targets CD20. (**c**) A glycoengineered antibody with a reduced fucose Fc region (purple), improving the antibody-dependent cellular cytotoxicity (ADCC). A notable example is obinutuzumab, which also targets CD20. (**d**) An antibody–drug conjugate with cytotoxic payload (red). A notable example is polatuzumab vedotin, which targets CD79b and delivers a payload of monomethyl auristatin E. (**e**) A bispecific antibody (shown as blue versus green) with distinct FAb complementarity. A notable example is glofitamab, which targets CD20 and CD3. Binding CD3 on a CD8+ T-cell activates its cytotoxic activity toward the approximated CD20+ cell. Adopted from Naman et al. [16].

**Figure 2:**
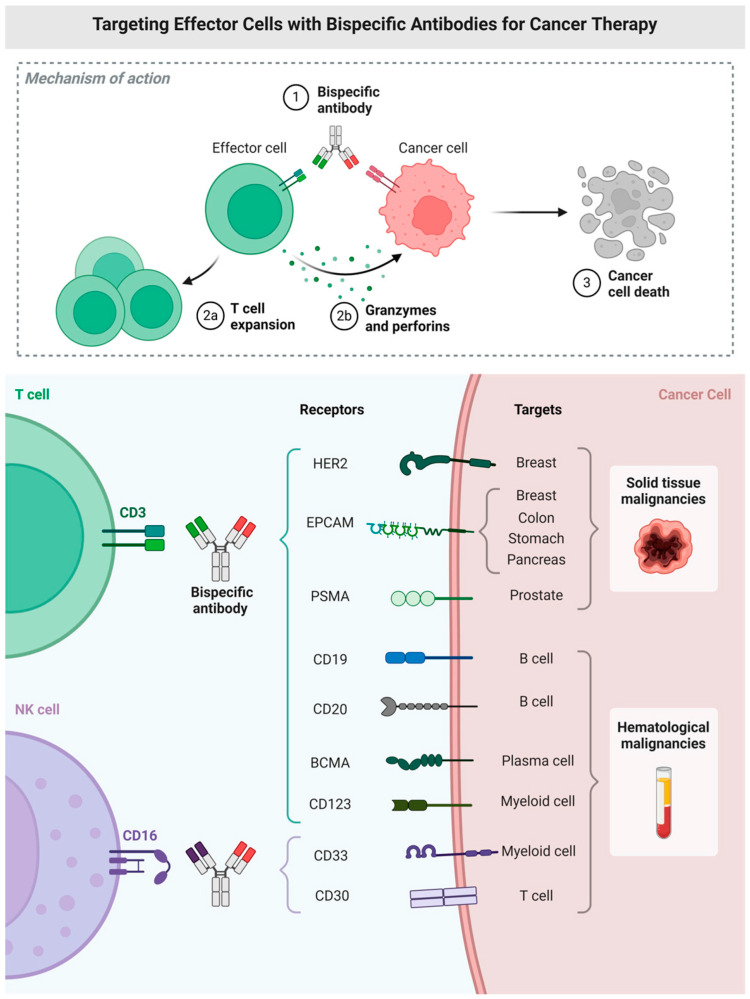
Targeting effector cells with bispecific antibodies for cancer therapy—illustrating mechanisms of T-cell and NK-cell redirection. Adopted from Amoozgar et al. [40].
